# A fibre optic oxygen sensor that detects rapid PO_2_ changes under simulated conditions of cyclical atelectasis *in vitro*^☆^

**DOI:** 10.1016/j.resp.2013.10.006

**Published:** 2014-01-15

**Authors:** Federico Formenti, Rongsheng Chen, Hanne McPeak, Martin Matejovic, Andrew D. Farmery, Clive E.W. Hahn

**Affiliations:** a Nuffield Division of Anaesthetics, Nuffield Department of Clinical Neurosciences, University of Oxford, John Radcliffe Hospital, Oxford OX3 9DU, UK; b Biomedical Centre, Charles University in Prague, Faculty of Medicine in Pilsen, alej Svobody 80, 304 60 Pilsen, Czech Republic; c First Medical Department, Charles University in Prague, Faculty of Medicine in Pilsen, alej Svobody 80, 304 60 Pilsen, Czech Republic

**Keywords:** Optical $P_{{\text{O}}_{2}}$ sensor, Cyclical atelectasis simulation, Cross-over computer control system

## Abstract

Two challenges in the management of Acute Respiratory Distress Syndrome are the difficulty in diagnosing cyclical atelectasis, and in individualising mechanical ventilation therapy in real-time. Commercial optical oxygen sensors can detect $P_{a_{{\text{O}}_{2}}}$ oscillations associated with cyclical atelectasis, but are not accurate at saturation levels below 90%, and contain a toxic fluorophore. We present a computer-controlled test rig, together with an in-house constructed ultra-rapid sensor to test the limitations of these sensors when exposed to rapidly changing $P_{{\text{O}}_{2}}$ in blood *in vitro*. We tested the sensors’ responses to simulated respiratory rates between 10 and 60 breaths per minute. Our sensor was able to detect the whole amplitude of the imposed $P_{{\text{O}}_{2}}$ oscillations, even at the highest respiratory rate. We also examined our sensor's resistance to clot formation by continuous *in vivo* deployment in non-heparinised flowing animal blood for 24 h, after which no adsorption of organic material on the sensor's surface was detectable by scanning electron microscopy.

## Highlights

• Real time detection of cyclical atelectasis is fundamental for individualised mechanical-ventilation therapy in ARDS.
• Intra-arterial oxygen sensors could be used to detect the breath-by-breath oscillations in PO_2_ during cyclical atelectasis.
• The fidelity with which oxygen sensors can detect these arterial PO_2_ oscillations depends on the sensors’ speed of response.
• We present a system for testing fast-response fibre optic oxygen sensors under simulated conditions of cyclical atelectasis.
• We show that a prototype fibre optic oxygen sensor, compatible with clinical use, can detect rapid PO_2_ changes *in vitro*.

## 1 Introduction

Two separate systems for the dynamic testing of both commercial and in-house constructed fast response time fibre optic oxygen sensors have been described recently. The first system (Saied et al., 2010) described a gas chamber apparatus for testing the time response and performance of fast commercial optical sensors in the gas phase; the second system (Chen et al., 2012b) described a hand-controlled fluid flow cross-over apparatus for testing fast response time fibre optic blood-gas sensors in the liquid phase.

We describe here a computer controlled system for simulating rapid breath-by-breath induced changes in arterial oxygen partial pressure $\left(P_{a_{{\text{O}}_{2}}}\right)$ in response to intra-breath changes in pulmonary shunt in the lung, which are induced by cyclical atelectasis, and for testing the capability of intravascular oxygen sensors to measure these fast changes in $P_{a_{{\text{O}}_{2}}}$ accurately. Cyclical atelectasis is a phenomenon in the lung whereby part of the lung collapses during expiration and then re-opens on inspiration (Duggan and Kavanagh, 2005). When the lung partly collapses, venous admixture occurs and the arterial blood $P_{{\text{O}}_{2}}$ falls, only to rise again as the lung opens up again on inspiration. This cyclical opening and collapsing of the lung during mechanical ventilation of the sick lung can cause further injury – known as Ventilator Induced Lung Injury (VILI) (Albert, 2012, Fan et al., 2013). The presence of oscillating $P_{a_{{\text{O}}_{2}}}$ may therefore serve as a physiologically important biomarker of cyclical atelectasis, and the design of an intravascular oxygen sensor to detect it presents a major diagnostic opportunity. Such sensors need to be more than an order of magnitude faster than the relatively slow, and now historic, electrochemical sensors that were first used to investigate cyclical atelectasis in an animal model (Williams et al., 2000).

In this new work, we describe a test rig specifically designed to mimic rapid switching on and off of pulmonary shunt in flowing blood, by switching two flowing blood supplies (with differing $P_{{\text{O}}_{2}}$) *in vitro* past both a new optical in-house designed $P_{a_{{\text{O}}_{2}}}$ sensor and a commercial sensor (Ocean Optics AL300) that has been used in recent cyclical atelectasis animal studies (Baumgardner et al., 2002). The same blood flow apparatus previously described in this journal was used (Chen et al., 2012b) (with updated oxygenators), but this time employing a computer controlled system to activate the switching of blood flows at varying duty cycles and simulated respiratory rates (RR).

Cyclic variations in the oxygenation of blood within the respiratory cycle were initially reported in 1961 (Bergman, 1961a, Bergman, 1961b). Several studies, presented and discussed in more detail in the discussion section, have explored the nature of these oscillations, especially in association with cyclical atelectasis in the lung, observed in the Acute Respiratory Distress Syndrome. Overall, these studies clearly indicate that very fast $P_{a_{{\text{O}}_{2}}}$ and SaO_2_ sensors are needed to follow, in real time, dynamic changes in arterial blood oxygen tension – and that a fast response blood-flow test apparatus is needed to ascertain if this new generation of optical oxygen sensors is fit for purpose. With this background in mind, we decided to modify the existing cross-over liquid flow apparatus (Chen et al., 2012b) to simulate cyclical pulmonary shunt changes with different *I*:*E* ratios and RRs. This would enable the in-house sensor, as well as the commercial Foxy AL 300 sensor, to be tested to examine if they had a fast enough time response to measure faithfully very fast oscillations in $P_{a_{{\text{O}}_{2}}}$ on-line in flowing blood, and to investigate if a diminution in $\Delta P_{a_{{\text{O}}_{2}}}$ with increasing RR could be due to sensor technology limitation or might be a true physiological phenomenon (Baumgardner et al., 2002). We also tested whether or not our in-house sensor was resistant to clot formation when exposed to flowing blood for a 24-h period *in vivo*.

## 2 Methods

### 2.1 The sensors

We investigated the capacity of an in-house, custom-built fibre optic $P_{{\text{O}}_{2}}$ sensor to detect rapid $P_{{\text{O}}_{2}}$ oscillations in blood *in vitro*. This sensor is made by coating the end section of a silica fibre with a Pt(II) doped polymer sensing material, poly(methyl methacrylate) (PMMA). This PMMA sensor is based on the principle of fluorescence quenching of the platinum complex by oxygen, and is compatible with clinical application. Further technical details about the sensor have been reported previously (Chen et al., 2012a). The Foxy-AL300 fibre optic $P_{{\text{O}}_{2}}$ sensor was used as a control for comparison with the PMMA sensor. Each sensor was calibrated in blood at 0 and 50 kPa before each experiment. The two sensors were connected to a phase measurement system (NeoFox, Ocean Optics, Dunedin, FL, USA), and interfaced to a computer through an A/D board sampling at 10 Hz (USB-6251, National Instruments, Austin, TX, USA). In order to compare our data with those reported in the literature (Baumgardner et al., 2002, Shi et al., 2011), the AL300 sensor was also connected to a light intensity measurement system (USB 2000 spectrometer, Ocean Optics, Dunedin, FL, USA), interfaced to a computer through the A/D board. Data were recorded on a computer by means of a custom program (LabView, National Instruments, Austin, TX, USA).

### 2.2 Blood flow rig

A flowing blood test system was used to generate rapid $P_{{\text{O}}_{2}}$ oscillations *in vitro*. Full technical details of this system have been presented in this journal (Chen et al., 2012b). Briefly, two standard medical paediatric oxygenators (Medos Hilite 1000LT, Medos Medizintechnik AG, Stolberg, Germany) were arranged to provide two parallel and independent extracorporeal circuits, where blood $P_{{\text{O}}_{2}}$ was maintained at 5 kPa (37 mmHg) or 50 kPa (375 mmHg), and $P_{{\text{CO}}_{2}}$ at 5 kPa (37 mmHg), and pH at 7.4. The $P_{{\text{O}}_{2}}$ reference values were confirmed through blood gas analysis (ABL710, Radiometer, Copenhagen, Denmark) for sensor calibration purposes and for monitoring before each experiment. Two peristaltic pumps maintained blood flow through the circuits. In order to simulate body temperature in a pig, sheep or lamb animal model, and to record data that are comparable with the published literature, blood temperature was maintained at 39 °C by circulating temperature-controlled water (Grant Instruments, Cambridge, UK) through the two oxygenators. Blood temperature was continuously monitored with a thermocouple (TES130, TES Electrical Electronic Corp., Taipei, Taiwan). Flow from either circuit was diverted alternately towards the sensor being tested by means of computer-controlled rapid switchover solenoid valves that exposed the sensor to abrupt blood $P_{{\text{O}}_{2}}$ changes. The frequency of the switchover was controlled by a PC together with a digital to analogue board (National Instruments USB-6251, National Instruments, Austin, TX, USA) and an electronic power switch, and was programmed to simulate RR of 10, 20, 30, 40, 50, and 60 bpm, with an inspired to expired (*I*:*E*) ratio of 1:1. For RR of 10 and 30 bpm, *I*:*E* ratios of 1:3 and 1:2, respectively were tested in order to investigate other clinically relevant conditions. Whole lambs’ blood (physiological temperature ∼39 °C) was collected from a local abattoir and heparinised immediately. Bench studies were conducted for a continuous period of 5 h.

### 2.3 PMMA sensor surface clotting

The PMMA in-house sensors were specifically tested over a 24 h period for anti-fouling properties in two separate non-heparinised *in vivo* animal studies. None of the sensors had any anticoagulant constituents embedded into their polymer materials (Chen et al., 2012a, Chen et al., 2012b), and since the animals (pigs, weight circa 38 kg) were non-heparinised, these conditions presented a realistic challenge to the sensors. The *in vivo* experiments were performed at the Faculty of Medicine, Charles University, Pilsen, Czech Republic. Experiments conformed to the National Institutes of Health Guidelines for the Use of Laboratory Animals, and the protocols were approved by the University Animal Care Committee. A total of four fibre optic sensors were tested: one sensor was deployed in a femoral artery and one in an ear vein in each of the two animals, to gather evidence of clot formation or other fouling. The animals were part of a separate study being performed at Charles University, Plzen, and the insertion and presence of the fibre optic sensors did not compromise those studies in any way. After intravascular deployment for 24 h, the sensors were removed, stored in a plastic tube and returned to Oxford for analysis.

Each sensor was examined by scanning electron microscopy (SEM) in Oxford, both in the unused state and after 24 h of continuous *in vivo* deployment. SEM Energy Dispersive X-ray (EDX) analysis was performed by means of a JEOL 6480 LV SEM equipped with an Oxford Instruments X-MAX80 SD X-ray detector and INCA X-ray analysis system. The analysis was performed using EDX, which investigates the characteristic X-rays produced by the interaction between the primary electron beam and the sample. The technique identifies all elements present with atomic numbers of 5 and greater (boron) with a detection limit of approximately 0.1 wt%. In this case the analysis was carried out in Low Vacuum mode with a gas pressure of 40 Pa (using air) to prevent charging on the uncoated samples.

### 2.4 Data analysis and statistics

Differences between experimental $\Delta P_{a_{{\text{O}}_{2}}}$ values were assessed statistically using ANOVA, followed by *post hoc* comparisons between conditions (IBM SPSS Statistics for Windows, Version 20.0; Armonk, NY, USA). Statistical significance was assumed at values of *p* < 0.05. Variables are presented as means ± SD, unless otherwise stated.

## 3 Results

### 3.1 Sensor's response to simulated respiratory rate

A PMMA sensor was tested for its response to the simulated RRs, together with an AL300 commercial sensor, over a five-hour period, at 39 °C. Because the blood in the test rig was heparinised, there were no concerns about blood clots forming on the sensor surface. The in-house PMMA and AL300 sensors were used to monitor continuous $\Delta P_{{\text{O}}_{2}}$ oscillations of 45 kPa peak-to-peak amplitude, from 5 kPa to 50 kPa (37–375 mmHg) at simulated respiratory rates from 10 to 60 bpm, over the five-hour period. Sensor output recording were taken at 20 min and 5 h during the experiments.

**Fig. 1:**
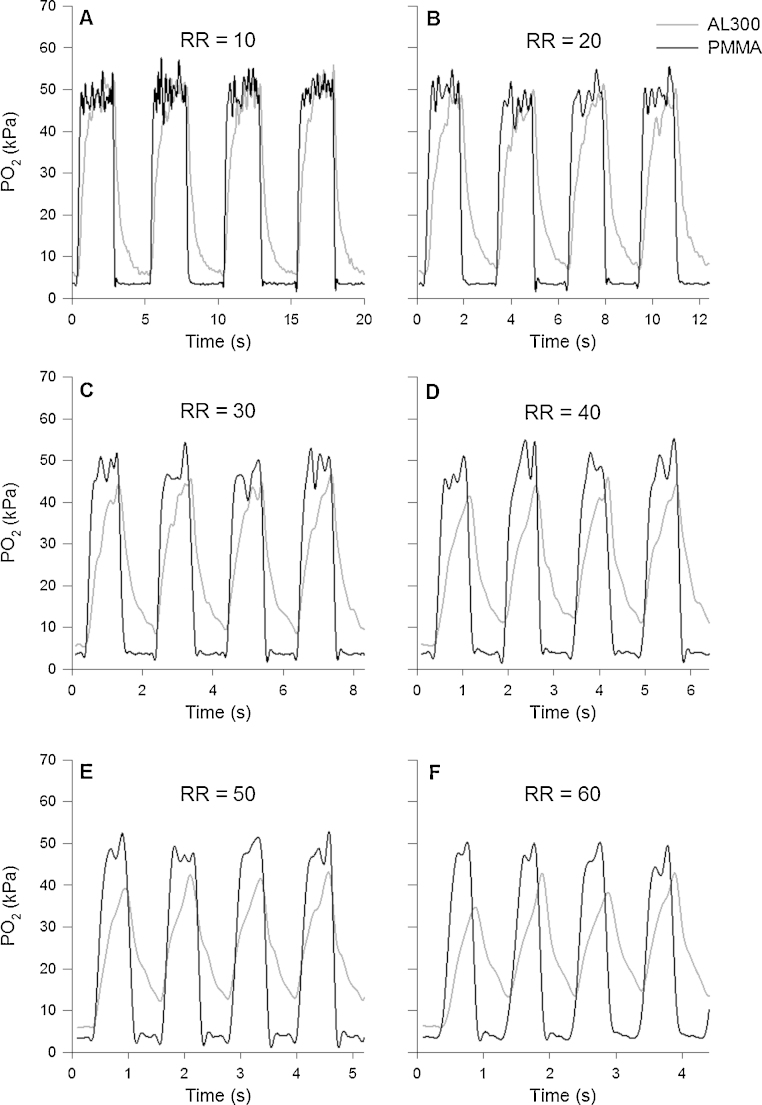
Synchronous amplitude-stable $P_{{\text{O}}_{2}}$ oscillations (5–50 kPa [37–375 mmHg]), recorded approximately 20 min after the sensors were immersed in flowing blood at 39 °C, are plotted against time (sensor up-date sampling rate: 10 Hz). The black and grey lines show data recorded by the PMMA and AL300 sensors respectively. The duty cycle was fixed at 50% to simulate an *I*:*E* ratio of 1:1. RR denotes respiratory rate. The PMMA sensor is seen to detect faithfully the entire $P_{{\text{O}}_{2}}$ oscillation amplitude at each RR, whereas the AL300 sensor fails to follow both the shape of the $P_{{\text{O}}_{2}}$ signal and its peak-to-peak amplitude.

Fig. 1 shows $P_{{\text{O}}_{2}}$ values recorded *in vitro* by both the PMMA and AL300 sensors in response to amplitude-stable $P_{{\text{O}}_{2}}$ oscillations at six simulated RRs in flowing blood at 39 °C. These values were recorded approximately 20 min after the sensors were immersed in blood. The response of the PMMA sensor was always faster than that of the AL300 sensor, and this was evident for all simulated RRs. In particular, the AL300 sensor demonstrated a different dynamic response to the increase and decrease in $P_{{\text{O}}_{2}}$, presenting a noticeably slower response (a long increasing and decreasing tail) in the detection of the $P_{{\text{O}}_{2}}$ change, especially when decreasing from 50 kPa to 5 kPa (375–37 mmHg). The PMMA sensor captured the whole of the 45 kPa (338 mmHg) $P_{{\text{O}}_{2}}$ step change even at the highest simulated RR (60 bpm); whereas the AL300 was able to record only 60% of the actual $P_{{\text{O}}_{2}}$ oscillation at 60 bpm.

**Fig. 2:**
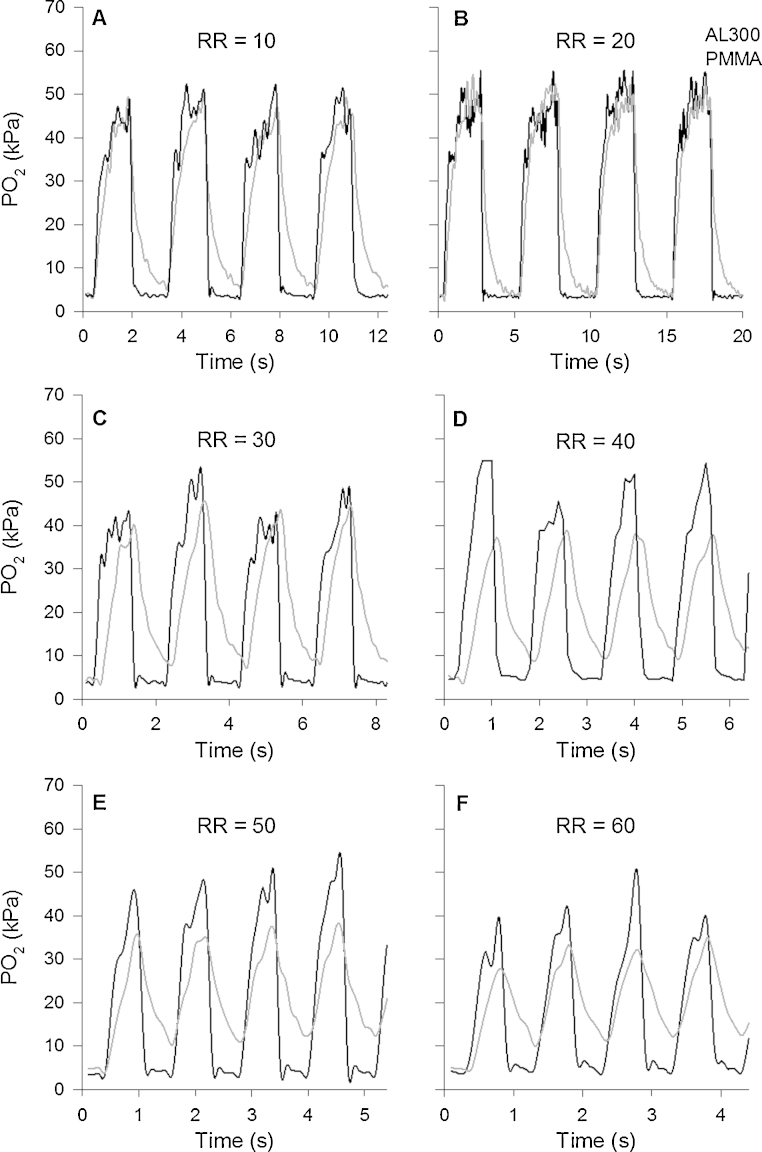
Synchronous amplitude-stable $P_{{\text{O}}_{2}}$ oscillations (5–50 kPa [37–375 mmHg]), recorded 5 h after both sensors were immersed in flowing blood at 39 °C, are shown as a function of time. Colour coding, sampling rate, and duty cycle are the same as in Fig. 1. The PMMA sensor is seen to detect most of the $P_{{\text{O}}_{2}}$ oscillation amplitude at each RR.

Similarly, Fig. 2 illustrates $P_{{\text{O}}_{2}}$ values recorded by the PMMA and AL300 sensors 5 h after they had been continuously immersed in flowing blood at 39 °C. The PMMA sensor still captured ∼90% of the 45 kPa (338 mmHg) $P_{{\text{O}}_{2}}$ step change, even at the highest simulated RR, where the AL300 sensor only captured ∼49% of the actual $P_{{\text{O}}_{2}}$ oscillation. The slow increasing and decreasing tails of the AL300 sensor are even more evident here as RR is increased.

**Fig. 3:**
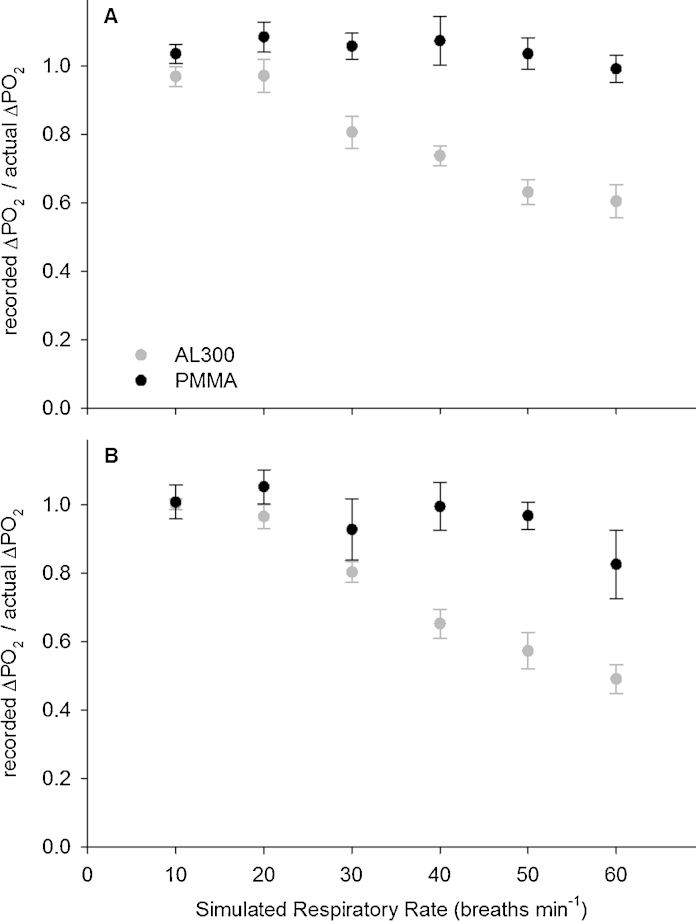
The recorded $P_{{\text{O}}_{2}}$ amplitude, expressed as a fraction of the actual value (recorded $\Delta P_{{\text{O}}_{2}}/\text{actual}\;\text{imposed}$$\Delta P_{{\text{O}}_{2}}$), in flowing blood at 39 °C is plotted against the simulated respiratory rate. The black and grey circles show data recorded by the PMMA and AL300 sensors respectively (average ± SD; oscillations *n* = 10). The duty cycle was fixed at 50%, simulating an *I*:*E* of 1:1. Panel A shows data recorded 20 min after the sensors were immersed in flowing blood, and panel B shows data recorded 5 h after continuous immersion.

Fig. 3A shows the relative $P_{{\text{O}}_{2}}$ oscillation amplitude (defined as $\Delta P_{{\text{O}}_{2}}$ recorded by the sensor, divided by the actual $\Delta P_{{\text{O}}_{2}}$ set by the test (i.e. 45 kPa [338 mmHg]) for the PMMA and the AL300 sensors, as a function of simulated RR in flowing blood at 39 °C. Twenty minutes after the sensors were immersed in blood, the PMMA sensor recorded the entire $P_{{\text{O}}_{2}}$ oscillation even at the highest RR (i.e. 60 bpm). The AL300 recorded the entire $P_{{\text{O}}_{2}}$ oscillation at the lowest RR, but it recorded smaller than actual $P_{{\text{O}}_{2}}$ oscillations as RR increased. The difference between the two sensors was statistically significant for each RR (*p* < 0.05).

Fig. 3B shows the values recorded after 5 h of continuous immersion in flowing blood at 39 °C. The PMMA sensor still recorded most of the actual $P_{{\text{O}}_{2}}$ oscillation at each RR, apart from at 60 bpm, where it recorded 83% of the actual $P_{{\text{O}}_{2}}$ oscillation. Five hours after immersion in flowing blood, the difference between the PMMA and AL300 sensors was statistically significant for RRs of 30, 40, 50, and 60 (*p* < 0.05).

### 3.2 Blood clotting

The surfaces of four PMMA sensors were free from deposits of organic material following insertion in the animal, non-heparinised, flowing blood for 24 h. The results of one sensor are shown below, but all four demonstrated the same apparent immunity from organic deposits.

**Fig. 4:**
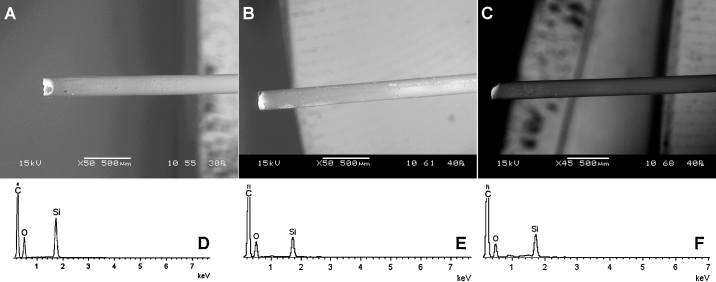
SEM images show the PMMA sensor (A) before immersion in blood, and 24 h after immersion in (B) arterial and (C) venous non-heparinised flowing animal blood. Spectra show relative quantity of elements detected (D) before *in vivo* insertion, and 24 h after continuous use in (E) arterial, and (F) venous blood. The elements detected did not appear different after immersion in blood, suggesting no clots had adhered to the sensors’ surface. C denotes carbon; O oxygen; and Si silicon, respectively.

Fig. 4 shows scanning electron microscopy (SEM) images of one PMMA sensor prior to insertion into the non-heparinised anaesthetised animal (Fig. 4A), and 24 h after continuous immersion in arterial (Fig. 4B) and venous blood (Fig. 4C). On a microscopic scale, there was no visible evidence of clotting on the sensors’ surfaces. Fig. 4D–F shows relative quantities of materials observed by EDX analysis on the surface of the sensors shown in Fig. 4A–C respectively. Carbon, silicon and oxygen were the elements predominantly detected (i.e. the component parts of the sensor's material itself). There was no apparent difference in observed elements between the clean and used sensors with respect to the carbon spectrum, indicating no adsorption of organic material.

## 4 Discussion

The apparatus presented here is shown to be capable of producing very fast continuous oscillations in $P_{{\text{O}}_{2}}$ over a 5-h period with freedom to vary simulated RR, *I*:*E* ratio and oscillation amplitude. Using temperature changes measured at the optical sensor site, it had been demonstrated previously that the switch-over of the two blood streams occurred within 50 ms at the sensor surface (Chen et al., 2012b), which is certainly fast enough to indicate that the mechanical switch-over of the two blood streams did not affect our results in any way. Any diminution in recorded $\Delta P_{{\text{O}}_{2}}$ with increasing simulated RR would therefore be due to sensor performance, rather than test rig limitation. Studies investigating cyclical atelectasis in the Acute Respiratory Distress Syndrome (ARDS), where $P_{{\text{O}}_{2}}$ varies widely within breaths, require very fast response intravascular oxygen sensors, which motivated the present study. $P_{{\text{O}}_{2}}$ and SaO_2_ oscillations in arterial blood have been studied for several decades; an overview of the most important findings in this field is presented and discussed in the following paragraph.

### 4.1 $P_{{\text{O}}_{2}}$ and SaO_2_ oscillations

Cyclic variations in blood oxygenation within the respiratory cycle were reported in 1961 in an open chest experimental animal model (Bergman, 1961a, Bergman, 1961b). In this model, femoral arterial blood was withdrawn from a small catheter through a fast response external oximetry cuvette at a constant rate by a motor-driven syringe, and variations in oxyhaemoglobin saturation (SaO_2_) were recorded in real time. SaO_2_ was used as a surrogate for arterial oxygen tension $\left(P_{{\text{O}}_{2}}\right)$, and rapid cyclic variations of up to 20% in SaO_2_ (ΔSaO_2_) were recorded. Using these saturation figures and a standard dissociation curve, these values translate to a $P_{{\text{O}}_{2}}$ oscillation amplitude of 15 mmHg at a mean $P_{{\text{O}}_{2}}$ of 36 mmHg (Whiteley et al., 2003). Despite the evidence suggesting that the cause of the observed fluctuations in arterial saturation might be due to variations in pulmonary shunt, it was concluded that these large variations in $P_{a_{{\text{O}}_{2}}}/{\text{SaO}}_{2}$ might be due to cyclical changes in alveolar oxygen tension. Much later on, in a computer model, it was shown that large changes in $P_{a_{{\text{O}}_{2}}}$ could only be generated by large intra-breath changes in pulmonary shunt caused, most likely, by cyclical atelectasis (Whiteley et al., 2003).

Oscillations in carotid artery $P_{{\text{O}}_{2}}$, which had the same period as respiration, were demonstrated in the cat, and in the newborn lamb in the first hours after birth (Purves, 1965, Purves, 1966). Although recognising that changes in venous admixture occur during the respiratory cycle and that there was a significant degree of venous admixture during the experiments, the conclusion was drawn that the cyclical oscillations in carotid $P_{{\text{O}}_{2}}\;(\Delta P_{a_{{\text{O}}_{2}}})$ in these animal studies were due to changes in alveolar $P_{{\text{O}}_{2}}$.

Thirteen years later, in an experimental cat model, it was shown that the amplitude of $\Delta P_{a_{{\text{O}}_{2}}}$ increased with increasing tidal volume, with increasing mean $P_{a_{{\text{O}}_{2}}}$, and decreasing ventilator frequency (Folgering et al., 1978). Some of these studies were conducted at a mean $P_{a_{{\text{O}}_{2}}}$ of 150 mmHg, i.e. on the “flat” part of the oxyhaemoglobin dissociation curve. $\Delta P_{a_{{\text{O}}_{2}}}$ varied from 45 mmHg to zero according to the mean $P_{a_{{\text{O}}_{2}}}$ experimental conditions and the chosen ventilator frequency. The miniature (1.2 mm diameter) intravascular $P_{a_{{\text{O}}_{2}}}$ sensors used in these studies were very specialised and were difficult for others to replicate – and so these experiments were not repeated by other workers.

Once a prototype intravascular $P_{{\text{O}}_{2}}$ sensor (IE Sensors, Salt Lake City, UT, USA) became available, investigations into cyclical $P_{a_{{\text{O}}_{2}}}$ oscillations in a lung lavage animal model of ARDS were performed (Williams et al., 2000). A large pulmonary shunt, typically 53%, was induced and $P_{a_{{\text{O}}_{2}}}$ oscillations were observed that were linked to the respiratory rate. The magnitude of the $P_{a_{{\text{O}}_{2}}}$ oscillations increased with applied positive end expiratory pressure (PEEP), and decreased when PEEP was reduced. The major failing in this study was that the prototype $P_{a_{{\text{O}}_{2}}}$ sensor had a slow response time, circa 5 s, and this slow response time severely attenuated the physiological oxygen signals. The study concluded that the most likely cause of the $\Delta P_{a_{{\text{O}}_{2}}}$ oscillations was cyclical atelectasis occurring in the animal's lungs, leading to a cyclical variation in pulmonary shunt as the lung opened and then closed during the inspiratory-expiratory cycle. The work was discontinued because the manufacturer ceased production of the prototype sensors.

### 4.2 Cyclical atelectasis and lung injury

Further studies investigating conditions such as volutrauma (stretch) and atelectrauma (cyclical recruitment) (Herweling et al., 2005, Otto et al., 2008, Syring et al., 2007) have confirmed the existence of $P_{a_{{\text{O}}_{2}}}$ oscillations that occur as possible mechanisms of ventilator–associated lung injury. Even more recent studies (Bodenstein et al., 2010, Hartmann et al., 2012, Shi et al., 2011) investigated the possibility of using SpO_2_ (oxygen saturation measured by pulse oximetry) oscillations (in parallel with $P_{a_{{\text{O}}_{2}}}$ oscillations) to detect the presence of cyclical atelectasis. These studies are new, but still employed a relatively slow oxygen sensing technology, and so no firm conclusions can be drawn as yet on the effect of elevated RRs on the amplitude of $P_{a_{{\text{O}}_{2}}}$ oscillations associated with cyclical atelectasis. A different explanation for $P_{a_{{\text{O}}_{2}}}$ oscillations that have the same period as breathing is related to regional aeration compartments and gas exchange in the lung, where pulmonary blood flow can cyclically be shifted from poorly to better ventilated regions in the lung (Gama de Abreu et al., 2010).

### 4.3 The effect of respiratory rate

The use of an ultra-fast (less than 1 s) ruthenium based fibre optic oxygen sensor (0.5 mm diameter), Ocean Optics AL300, and of a lung lavage rabbit model of ARDS highlighted the importance of RR in the mechanical ventilator management (Baumgardner et al., 2002). Although the sensor used in this study was not designed for animal *in vivo* use, it was adequate enough to capture most of the large and rapid $\Delta P_{a_{{\text{O}}_{2}}}$, which ranged up to 334 or 439 mm Hg. These results indicated that RR, PEEP and plateau pressure minus PEEP all had significant effects on the magnitude of $\Delta P_{a_{{\text{O}}_{2}}}$, but that RR and PEEP were much more significant predictor values. As with previous studies (Folgering et al., 1978, Purves, 1965, Purves, 1966), this work was conducted on the flat part of the dissociation curve (the rabbits inspired 100% oxygen), where small changes in arterial oxygen content (or saturation) would lead to relatively large changes in $P_{a_{{\text{O}}_{2}}}$. In agreement with conclusions previously reported in the literature (Williams et al., 2000), this study concluded that the large $P_{a_{{\text{O}}_{2}}}$ oscillations suggested significant cyclic recruitment of atelectasis in the animal surfactant depletion model.

The need for very fast oxygen and saturation sensors became clearer when $\Delta P_{a_{{\text{O}}_{2}}}$ appeared to be linked to RR in studies of ARDS animal models (Baumgardner et al., 2002, Folgering et al., 1978, Hartmann et al., 2012, Shi et al., 2011, Syring et al., 2007). Taken together, RR was varied between 6 bpm and 30 bpm in these animal studies, where RRs greater than 20 bpm were generally associated with reduced $P_{a_{{\text{O}}_{2}}}$ oscillation amplitude (from ∼26 to 2.6 kPa [∼200–20 mmHg]), especially when no or low PEEP was applied. This decrease in the amplitude of $P_{a_{{\text{O}}_{2}}}$ oscillations was attributed to the effect of high RRs on maintaining lung recruitment, yet it appeared unclear whether this result was a physiological phenomenon or, possibly, a failure of the AL300 sensor to respond fast enough to catch the true magnitude of the physiological oscillations at high RRs. In fact, it was calculated that the AL300 sensor would detect only about 80% of the actual $P_{a_{{\text{O}}_{2}}}$ oscillation at RR of 24 bpm, and thus presumably smaller proportions at higher RRs (Costa and Amato, 2007); this inaccuracy in the $P_{a_{{\text{O}}_{2}}}$ measurements is acceptable in terms of maintenance of end-expiratory recruitment up to RRs of about 20 bpm (Baumgardner and Syring, 2007).

### 4.4 Our results

Fig. 1, Fig. 2, Fig. 3 confirm the AL300 sensor's incapacity to measure large $P_{{\text{O}}_{2}}$ oscillations at elevated RR *in vitro* (on the test rig), where no effect can be attributed to lung recruitment. The question of whether or not the diminutions in the recorded rabbit $\Delta P_{a_{{\text{O}}_{2}}}$ with increasing RR are due to physiology or diminution in sensor performance (or a mixture of both) still remains unresolved, and the physiological implications for the AL300's limited accuracy at RR of ∼30 bpm or greater are unclear. However, it seems clear that the fastest possible $P_{a_{{\text{O}}_{2}}}$ sensor should be used to provide more reliable information at any RR, including high RRs between 30 bpm and 60 bpm. This would then afford the opportunity to extend the use of this sensing technology to neonatal intensive care units and small animal studies.

Apart from being able to detect the true amplitude peaks of the $P_{{\text{O}}_{2}}$ oscillations, the in-house PMMA sensor produced a more faithful dynamic response throughout the $P_{{\text{O}}_{2}}$ transitions, in particular during the simulated expiratory phase (i.e. the decrease in $P_{{\text{O}}_{2}}$, as seen in Fig. 1, Fig. 2). This phenomenon was observed at all RR and *I*:*E* ratios, including *I*:*E* ratios of 1:3 and 1:2 (data not shown, but recorded in our studies). In critical care settings, the PMMA sensor's fast response time could offer the possibility to detect the kinetics of lung collapse more accurately, and to monitor the effects of lung recruiting manoeuvres on a breath-by-breath basis. In a wider perspective, it could provide information on the kinetics of alveolar recruitment, the understanding of which might form the basis of attempts to moderate the risks of ventilation-induced lung injury (Albert, 2012), and to support the development of new mathematical models of the lung (Hahn and Farmery, 2003, Suki et al., 1994, Whiteley et al., 2003).

A comment can also be made here on the limitations of the technology used by the AL300 sensor. The fluorescence *intensity* measurement (Baumgardner et al., 2002, Syring et al., 2007) is not only a function of the local $P_{{\text{O}}_{2}}$, but it also depends on the optical properties of the medium, the ambient light intensity and potential degradation of the sensor fluorophore itself (McDonagh et al., 2001). Some fluorescence will be transmitted directly down the fibre to be measured, and a variable amount of light will be scattered by the red blood cells before being transmitted back down the fibre. This scattered light intensity will vary with haematocrit and with the colour (i.e. saturation) of the blood, meaning that the signal is also influenced by SaO_2_. Light intensity dependent sensors must be calibrated uniquely for each clinical setting, and their output will be somewhat non-linear.

In particular, intensity measurement could become particularly inaccurate when saturation drops below ∼90%, where relatively small changes in $P_{{\text{O}}_{2}}$ are associated with large changes in saturation. Because of this limitation, it is not possible to compare directly $P_{a_{{\text{O}}_{2}}}$ oscillations and varying shunt fraction for oxygen saturation levels below 90%. In order to avoid this technical limitation, previous studies [apart from Bergman, 1961a, Bergman, 1961b] have restricted their ARDS animal models to small shunts (where arterial blood saturation was maintained near to 100%) and so changes in saturation did not influence the measurements (Baumgardner et al., 2002, Syring et al., 2007). This, however, is not entirely reflective of the population of patients in the critical care setting who may have more significant degrees of recruitable and non-recruitable shunt and who may be desaturated throughout the respiratory cycle, or at least at end-expiration. An alternative solution is to measure fluorescence quenching lifetime (McDonagh et al., 2001), where the measured signal is independent of the optical properties of the medium, including blood in the AL300 sensor oxygen saturation; with this approach, our in-house sensor is capable of measuring $P_{{\text{O}}_{2}}$ independently of haemoglobin saturation. Having said that, we did not find a marked difference in measured $P_{{\text{O}}_{2}}$ in the AL300 sensor, when we compared values calculated from fluorescence intensity (data not shown) with values from fluorescence quenching time constant measurements. This result was most likely observed because our two calibration points (peak and trough) were exactly the values that we subsequently measured. It is unlikely that any values in between would be accurately calibrated, which highlights the fact that sensors based on intensity measurement need to be calibrated specifically for the ranges and conditions in which they are intended to be used.

A second potential limitation of any intravascular oxygen sensing is that *in vivo* sensors are prone to biofouling with adsorbed material such as fibrin or large clots, which would impair the signal recorded by the sensor. This is a long recognised problem with intravascular sensors (Severinghaus and Astrup, 1986). In this respect, all four of our in-house PMMA sensors remained free from clotting after continuous immersion in non-heparinised flowing blood for a period of 24 h (see Fig. 4). This lack of clotting on the surface of the PMMA sensor suggests that it would be capable of measuring $P_{a_{{\text{O}}_{2}}}$ oscillations at least for a 24-h period, a much longer period than that considered in previous studies.

Our results demonstrate that the commercial AL300 fibre optic oxygen sensor currently used in animal research has a relatively slow response time for the detection of rapid $P_{a_{{\text{O}}_{2}}}$ oscillations, and would not be accurate at varying levels of oxygen saturations or high RR. Furthermore, it is made with ruthenium, a toxic material that is reported to be unsafe in the clinical setting (Yasbin et al., 1980). It is currently unknown whether the AL300 sensor is resistant to clotting when challenged with continuous immersion in whole blood for a period of 24 h, hence it is unknown how immersion in blood for this duration of time may affect its performance. In contrast, the in-house PMMA sensor demonstrates that faster oxygen sensing technology is now available made of materials suitable for clinical application, and resistant to clotting for at least 24 h.

The apparatus that we have described here is also suitable to be used with fast time response SaO_2_ sensors, if and when they are constructed, or with any other intravascular pH or CO_2_ sensor.

## Funding

The laboratory and animal work was supported by a Wellcome Trust Translation Award, Wellcome Trust, UK.
